# Spectral properties of a class of unicyclic graphs

**DOI:** 10.1186/s13660-017-1367-2

**Published:** 2017-05-03

**Authors:** Zhibin Du

**Affiliations:** School of Mathematics and Statistics, Zhaoqing University, Zhaoqing, 526061 China

**Keywords:** 05C50, 15A42, spectral radius, least eigenvalue, spread, unicyclic graphs

## Abstract

The eigenvalues of *G* are denoted by $\lambda_{1}(G), \lambda_{2}(G), \ldots, \lambda_{n}(G)$, where *n* is the order of *G*. In particular, $\lambda _{1}(G)$ is called the spectral radius of *G*, $\lambda_{n}(G)$ is the least eigenvalue of *G*, and the spread of *G* is defined to be the difference between $\lambda_{1}(G)$ and $\lambda_{n}(G)$. Let $\mathbb{U}(n)$ be the set of *n*-vertex unicyclic graphs, each of whose vertices on the unique cycle is of degree at least three. We characterize the graphs with the *k*th maximum spectral radius among graphs in $\mathbb{U}(n)$ for $k=1$ if $n\ge6$, $k=2$ if $n\ge8$, and $k=3,4,5$ if $n\ge10$, and the graph with minimum least eigenvalue (maximum spread, respectively) among graphs in $\mathbb{U}(n)$ for $n\ge6$.

## Introduction

Let *G* be a simple graph with vertex set $V(G)$ and edge set $E(G)$. Let ${\mathbf{A}}(G)$ be the adjacency matrix of *G*. The characteristic polynomial of *G* is the characteristic polynomial of ${\mathbf{A}}(G)$, denoted by $\phi(G,x)$. The eigenvalues of *G*, denoted by $\lambda_{1}(G), \lambda_{2}(G), \ldots, \lambda_{n}(G)$, are the eigenvalues of ${\mathbf{A}}(G)$, arranged in a non-increasing order, where *n* is the order of *G*.

In particular, $\lambda_{1}(G)$ is called the spectral radius of *G*, and we usually use $\lambda_{n}(G)$ to represent the least eigenvalue of *G*. The spread of *G*, denoted by $s(G)$, is the difference between the largest and the least eigenvalues, *i.e.*, $s(G)=\lambda_{1}(G)-\lambda_{n}(G)$.

By the Perron-Frobenius theorem [1], for a connected graph *G*, corresponding to $\lambda_{1}(G)$, there is a unit eigenvector ${\mathbf{x}}=(x_{1},x_{2},\ldots, x_{n})^{T}$ with all positive entries, known as the principal eigenvector of *G*. For a connected graph *G*, it is well known that [1]

$$\lambda_{1}(G)\ge-\lambda_{n}(G) $$

with equality if and only if *G* is bipartite.

A unicyclic graph is a connected graph with a unique cycle. The spectral radius of unicyclic graphs has been studied extensively these years. Guo [2] determined the graphs with the first ten maximum spectral radii among all the *n*-vertex unicyclic graphs for $n\ge17$. Belardo *et al.* [3] determined the maximum spectral radius of unicyclic graphs with given girth. Yu and Tian [4] gave the first two spectral radii of unicyclic graphs with a given matching number. More results on the spectral radius of unicyclic graphs can be found in [5–7].

The least eigenvalue of graphs is a relatively new topic in spectral graph theory. Fan *et al.* [8] characterized the graph with minimum least eigenvalue (the graph with maximum spread, respectively) among all the *n*-vertex unicyclic graphs for $n\ge4$. Zhai *et al.* [9] determined the minimum least eigenvalue of unicyclic graphs with given diameter. Du [10] presented the first six minimum least eigenvalues of unicyclic graphs. This paper will continue the research of the spectral properties of unicyclic graphs, such as spectral radius, the least eigenvalue, and the spread.

A unicyclic graph is called fully loaded if every vertex on its unique cycle has degree at least three. Let $\mathbb{U}(n)$ be the set of *n*-vertex fully loaded unicyclic graphs, where $n\ge6$. In this paper, we characterize the graphs with the *k*th maximum spectral radius among graphs in $\mathbb{U}(n)$ for $k=1$ if $n\ge6$, $k=2$ if $n\ge8$, and $k=3,4,5$ if $n\ge10$, and the graph with minimum least eigenvalue (maximum spread, respectively) among graphs in $\mathbb{U}(n)$ for $n\ge6$.

## Preliminaries

We use standard notation from spectral graph theory [1].

Let **x** be a unit eigenvector of *G* corresponding to $\lambda_{1}(G)$ or $\lambda_{n}(G)$. We say $x_{v}$ is the element of **x** corresponding to $v\in V(G)$.

### Lemma 1

[3, 11]

*Let* *G* *be a connected graph*, $rs\in E(G)$ *and* $rt\notin E(G)$. *Let* $G'$ *be the graph obtained from* *G* *by deleting the edge* *rs* *and adding the edge* *rt*. *Let* **x** *and* ${\mathbf{x}}'$ *be*, *respectively*, *the principal eigenvectors of* *G* *and* $G'$. *If* $x_{t}\ge x_{s}$, *then* $\lambda_{1}(G')>\lambda_{1}(G)$ *and* $x_{t}'>x_{s}'$.

For $u\in V(G)$, let $d_{G}(u)$ be the degree of *u* in *G*. A pendent vertex is a vertex of degree one.

### Lemma 2

[12]

*Let* *G* *be a connected graph with* $uv\in E(G)$ *and* $d_{G}(u),d_{G}(v)\ge 2$. *Suppose that* *u* *and* *v* *have no common neighbor*. *Let* $G'$ *be the graph obtained from* *G* *by deleting the edge* *uv* *and identifying* *u* *and* *v*, *which is denoted by* *w*, *and attaching a pendent vertex to* *w*. *Then* $\lambda_{1}(G')>\lambda_{1}(G)$.

For a vertex subset $V'$ of *G*, let $G-V'$ denote the graph formed from *G* by deleting all the vertices in $V'$ and their incident edges. In particular, we write $G-u$ for $G-\{u\}$.

### Lemma 3

[1]

*Let* *v* *be a vertex of a graph* *G* *different from a cycle*, *and let* $\varphi(v)$ *be the set of the cycles containing* *v*. *Then*

$$\phi(G,x)=x\cdot\phi(G-v,x)-\sum_{vw\in E(G)} \phi(G-v-w,x)-2\sum_{Z\in\varphi(v)}\phi\bigl(G-V(Z),x\bigr). $$

*In particular*, *if* *v* *is a pendent vertex of* *G* *with unique neighbor* *u*, *then*

$$\phi(G,x)=x\cdot\phi(G-v,x)-\phi(G-v-u,x), $$

*where* $\phi(G-v-u,x)=1$ *if* $G=P_{2}$.

### Lemma 4

[11, 13]

*Let* *G* *be a connected non*-*trivial graph*, *and let* *H* *be a proper spanning subgraph of* *G*. *Then* $\phi(H,x)>\phi(G,x)$ *for* $x\ge\lambda_{1}(G)$.

### Lemma 5

*Let* $G_{0}$ *be a connected graph with at least three vertices*, *where* $u,v \in V(G_{0})$, *and let* $H_{0}$ *be a connected graph with* $w\in V(H_{0})$. *Let* $G_{1}$ ($G_{2}$, *respectively*) *be the graph obtained from* $G_{0}$ *and* $H_{0}$ *by identifying* *u* (*v*, *respectively*) *with* *w*. *Let* **y** ($\mathbf{y}'$, *respectively*) *be a unit eigenvector of* $G_{1}$ ($G_{2}$, *respectively*) *corresponding to* $\lambda _{n}(G_{1})$ ($\lambda_{n}(G_{2})$, *respectively*). *Suppose that* $|y_{u}| \le|y_{v}|$.

(i) [8] $\lambda_{n}(G_{1})\ge\lambda_{n}(G_{2})$ *with equality if and only if* $y_{u}= y_{v}$ *and* $\sum y_{j}= 0$, *where the summation takes over all the neighbors of* *w* *in* $H_{0}$.
(ii) *If* $\lambda_{n}(G_{1})> \lambda_{n}(G_{2})$, *then* $|y_{u}'| < |y_{v}'|$.

### Proof

We need only to prove (ii). If $|y_{u}'|\ge|y_{v}'|$, then by (i), $\lambda_{n}(G_{1})\le\lambda_{n}(G_{2})$, which is a contradiction. □

By Lemma 5(i), we have the following.

### Lemma 6

[8]

*Let* *u* *be a vertex of a connected graph* *Q* *with at least two vertices*. *For integer* $a\ge1$, *let* $G_{1}$ *be the graph obtained by attaching a star* $S_{a+1}$ *at its center* *v* *to* *u* *of* *Q*, *and* $G_{2}$ *be the graph obtained by attaching* $a+1$ *pendent vertices to* *u* *of* *Q*. *Then* $\lambda_{n}(G_{1})\ge\lambda_{n}(G_{2})$.

For an edge subset *M* of *G*, let $G-M$ denote the graph obtained from *G* by deleting the edges in *M*, and for an edge subset $M^{*}$ of the complement of *G*, let $G+M^{*}$ denote the graph obtained from *G* by adding the edges in $M^{*}$.

## Large spectral radius of graphs in $\mathbb{U}(n)$

In this section, we determine the first five maximum spectral radii of graphs in $\mathbb{U}(n)$.

Let $U_{n,m}$ be the unicyclic graph obtained from the cycle $C_{m}=v_{1}v_{2}\cdots v_{m}v_{1}$ by attaching $n-2m+1$ pendent vertices to $v_{1}$ and a pendent vertex to each of the other vertices on $C_{m}$. Obviously, $U_{n,m}\in\mathbb{U}(n)$.

### Lemma 7

*Let* $G\in\mathbb{U}(n)$ *with unique cycle of length* $m \ge3$, *where* $n \ge2m$. *Then* $\lambda_{1}(G)\le\lambda_{1}(U_{n,m})$ *with equality if and only if* $G \cong U_{n,m}$.

### Proof

Let *G* be a graph with maximum spectral radius satisfying the given condition. Let $C_{m}=v_{1}v_{2}\cdots v_{m}v_{1}$ be the unique cycle of *G*. By Lemma 2, the vertices of *G* outside $C_{m}$ are all pendent vertices.

Suppose that $G\ncong U_{n,m}$. Then we may choose two vertices, say $v_{i}$ and $v_{j}$, on $C_{m}$ such that $d_{G}(v_{i}),d_{G}(v_{j})\ge4$, where $1\le i < j\le m$. Let **x** be the principal eigenvector of *G*. Suppose without loss of generality that $x_{v_{i}}\ge x_{v_{j}}$. Consider $G_{1}=G-vv_{j}+vv_{i}$, where *v* is a neighbor of $v_{j}$ outside $C_{m}$. Obviously, $G_{1}\in\mathbb{U}(n)$ and its unique cycle is still of length *m*. By Lemma 1, $\lambda_{1}(G)<\lambda_{1}(G_{1})$, which is a contradiction. Thus there is at most one vertex on $C_{m}$ with degree at least four in *G*, *i.e.*, $G \cong U_{n,m}$. □

### Lemma 8

*Let* $G\in\mathbb{U}(n)$ *with unique cycle of length* $m \ge4$, *where* $n\ge2m$. *Then* $\lambda_{1}(G)\le\lambda_{1}(U_{n,4})$ *with equality if and only if* $G \cong U_{n,4}$.

### Proof

If $m\ge5$, then applying Lemma 2 to $G=U_{n,m}$ by setting *uv* to be an edge on the cycle incident to the vertex of maximum degree, we have $\lambda_{1}(U_{n,m})<\lambda_{1}(U_{n,m-1})$, and thus $\lambda_{1}(U_{n,m})<\lambda_{1}(U_{n,4})$. If $m=4$, then by Lemma 7, $\lambda_{1}(G)\le\lambda_{1}(U_{n,4})$ with equality if and only if $G \cong U_{n,4}$. □

Let $C_{3}(T_{1},T_{2},T_{3})$ be the *n*-vertex unicyclic graph with the triangle $v_{1}v_{2}v_{3}v_{1}$ such that the deletion of the three edges on the triangle would result in three vertex-disjoint trees $T_{1}$, $T_{2}$, $T_{3}$, where $v_{i}\in V(T_{i})$ for $i=1,2,3$.

Let $S_{n}(a,b,c)=C_{3}(T_{1},T_{2},T_{3})$, where $|V(T_{1})|=a-1$, $|V(T_{2})|=b-1$, $|V(T_{3})|=c-1$, $a+b+c=n+3$, $a\ge b\ge c\ge2$, and $T_{1}$, $T_{2}$, $T_{3}$ are all stars with centers $v_{1}$, $v_{2}$, $v_{3}$, respectively. Obviously, $S_{n}(n-3,3,3) \cong U_{n,3}$.

### Lemma 9

*Among the graphs* $S_{n}(a,b,c)$ *with* $a+b+c=n+3$ *and* $a\ge b\ge c\ge 3$, $S_{n}(n-3,3,3) \cong U_{n,3}$ *for* $n\ge6$, $S_{n}(n-4,4,3)$ *for* $n\ge 8$, *and* $S_{n}(n-5,5,3)$ *for* $n\ge10$ *are*, *respectively*, *the unique graphs with the first*, *the second*, *and the third maximum spectral radii*.

### Proof

Let **x** be the principal eigenvector of $S_{n}(a,b,c)$.

Suppose that $a>b$. If $x_{v_{1}}< x_{v_{2}}$, then by Lemma 1,

$$\lambda_{1}\bigl(S_{n}(a,b,c)\bigr)< \lambda_{1} \bigl(S_{n}(a-1,b+1,c)\bigr)< \cdots< \lambda_{1} \bigl(S_{n}(b,a,c)\bigr), $$

which is a contradiction. If $x_{v_{1}}\ge x_{v_{2}}$, then by Lemma 1,

$$\lambda_{1}\bigl(S_{n}(a,b,c)\bigr)< \lambda_{1} \bigl(S_{n}(a+1,b-1,c)\bigr). $$

If $a=b$, then whether $x_{v_{1}}\ge x_{v_{2}}$ or $x_{v_{1}}< x_{v_{2}}$, we have by Lemma 1 that

$$\lambda_{1}\bigl(S_{n}(a,b,c)\bigr)< \lambda_{1} \bigl(S_{n}(a+1,b-1,c)\bigr). $$

Then

$$\lambda_{1}\bigl(S_{n}(a,b,c)\bigr)< \lambda_{1} \bigl(S_{n}(a+1,b-1,c)\bigr) $$

for $b\ge4$. This implies that among the graphs $S_{n}(a,b,3)$ with $a+b+3=n+3$ and $a\ge b\ge3$, $S_{n}(n-3,3,3) \cong U_{n,3}$ for $n\ge6$, $S_{n}(n-4,4,3)$ for $n\ge8$, and $S_{n}(n-5,5,3)$ for $n\ge10$ are, respectively, the unique graphs with the first, the second, and the third maximum spectral radius.

If $c\ge4$, then by similar arguments as above,

$$\lambda_{1}\bigl(S_{n}(a,b,c)\bigr)\le \lambda_{1}\bigl(S_{n}(n-5,4,4)\bigr)< \lambda_{1} \bigl(S_{n}(n-5,5,3)\bigr). $$

Now the result follows. □

Let $U_{n}^{1}$ be the unicyclic graph obtained by attaching a path on two vertices to the vertex of degree $n-5$ of $S_{n-2}(n-5,3,3)$, where $n\ge8$. Let $U_{n}^{2}$ be the unicyclic graph obtained by attaching a path on two vertices to the vertex of degree two of $S_{n-2}(n-4,3,2)$, where $n\ge7$. Let $U_{n}^{3}$ be the unicyclic graph obtained by attaching a path on two vertices to the vertex of degree $n-6$ of $S_{n-2}(n-6,4,3)$, where $n\ge10$.

For $u,v\in V(G)$, let $d_{G}(u,v)$ be the distance from *u* to *v* in *G*.

Let $P_{n}$ be the path on *n* vertices. Let $G \cup H$ be the vertex-disjoint union of graphs *G* and *H*.

### Lemma 10

*Let* $G=C_{3}(T_{1},T_{2},T_{3})$ *with* $|V(T_{1})|=a-1$, $|V(T_{2})|=b-1$, $|V(T_{3})|=2$, $a+b=n$, $a\ge b$, $b=3, 4$, *and* $n\ge 10$. *Suppose that* $G\ncong S_{n}(a,b,c), U_{n}^{1}, U_{n}^{2}$. *Then* $\lambda_{1}(G)<\lambda_{1}(S_{n}(n-5,5,3))$.

### Proof

First suppose that $b=3$. Let $r=\max\{d_{G}(u,v_{1}):u\in V(T_{1})\}$. Obviously, $r\ge2$ as $G\ncong S_{n}(a,b,c)$.

If $r\ge3$, then by Lemma 2, we may get a unicyclic graph with $r=2$ with a larger spectral radius.

Suppose that $r=2$.

If there are at least two non-pendent neighbors of $v_{1}$ outside the triangle in *G*, then by Lemma 2, we may get a unicyclic graph with exactly one non-pendent neighbor of $v_{1}$ outside the triangle with a larger spectral radius.

Suppose that there is exactly one non-pendent neighbor, say *w*, of $v_{1}$ outside the triangle of *G*. Let $d_{G}(v_{1})=s$ and $d_{G}(w)=t$. Obviously, $s\ge3$, and $t\ge3$ as $G\ncong U_{n}^{1}$. If $t=3$, then *G* is the graph obtained by attaching a pendent vertex to the vertex of degree two in $U_{n-1}^{1}$, which is denoted by $L_{n}$. If $t=n-5$, then *G* is the graph obtained by attaching $n-6$ pendent vertices to a pendent vertex of $S_{6}(3,3,3)$, which is denoted by $H_{n}$.

Suppose that $4\le t\le n-6$. Let **x** be the principal eigenvector of *G*. If $x_{w}\ge x_{v_{1}}$, then by Lemma 1, $\lambda_{1}(G)<\lambda_{1}(H_{n})$. If $x_{w}< x_{v_{1}}$, then by Lemma 1, $\lambda_{1}(G)<\lambda_{1}(L_{n})$. Thus

$$\lambda_{1}(G)< \max\bigl\{ \lambda_{1}(L_{n}), \lambda_{1}(H_{n})\bigr\} . $$

Now we compare $\lambda_{1}(L_{n})$ with $\lambda_{1}(H_{n})$. Let $r_{n}(x)=\phi(L_{n},x)-\phi(H_{n},x)$. Applying Lemma 3 to $G=L_{n}$ by setting *v* to be a pendent neighbor of $v_{1}$,

$$\phi(L_{n},x)=x\cdot\phi(L_{n-1},x)-x^{n-9} \phi(P_{3}\cup P_{4},x), $$

and to $G=H_{n}$ by setting *v* to be a pendent vertex whose unique neighbor is of degree $n-5$,

$$\phi(H_{n},x)=x\cdot\phi(H_{n-1},x)-x^{n-7}\phi \bigl(S_{5}(3,3,2),x\bigr), $$

which implies that

$$\begin{aligned} r_{n}(x) =&x\cdot r_{n-1}(x)+x^{n-7}\phi \bigl(S_{5}(3,3,2),x\bigr)-x^{n-9}\phi (P_{3}\cup P_{4},x) \\ =&x\cdot r_{n-1}(x)+x^{n-7}\bigl(x^{5}-5x^{3}-2x^{2}+3x \bigr) \\ &{}-x^{n-9}\bigl(x^{3}-2x\bigr) \bigl(x^{4}-3x^{2}+1 \bigr) \\ =&x\cdot r_{n-1}(x)+2x^{n-8}\bigl(1-2x^{2}-x^{3} \bigr). \end{aligned}$$

Note that $r_{8}(x)=0$ as $L_{8}=H_{8}$, and it is easily seen that $1-2x^{2}-x^{3}<0$ for $x>1$, and thus $r_{n}(x)<0$ (*i.e.*, $\phi(L_{n},x)<\phi(H_{n},x)$) for $x>1$, which implies that $\lambda_{1}(L_{n})>\lambda_{1}(H_{n})$. Then

$$\lambda_{1}(G)\le \lambda_{1}(L_{n}) $$

if $b=3$.

Suppose that $b=4$. First suppose that $d_{G}(v_{2})=3$. Note that $G\ncong U_{n}^{2}$, then by similar arguments as above, $\lambda_{1}(G)\le \lambda_{1}(H)$, where *H* is the graph obtained by attaching a path on two vertices to the vertex of degree $n-6$ in $U_{n-2}^{2}$. By Lemma 2, $\lambda_{1}(H)<\lambda_{1}(U_{n}^{3})$. If $d_{G}(v_{2})=4$, then by similar arguments as above, $\lambda_{1}(G)\le \lambda_{1}(U_{n}^{3})$. Thus

$$\lambda_{1}(G)\le\lambda_{1}\bigl(U_{n}^{3} \bigr) $$

if $b=4$.

Now we have shown that

$$\lambda_{1}(G)\le \max\bigl\{ \lambda_{1}(L_{n}), \lambda_{1}\bigl(U_{n}^{3}\bigr)\bigr\} $$

for $b=3\mbox{ or }4$.

To determine $\max\{\lambda_{1}(L_{n}),\lambda_{1}(U_{n}^{3})\}$, we compare $\lambda_{1}(U_{n}^{3})$ with $\lambda_{1}(L_{n})$. Let $T^{(1)}$ be the $(n-3)$-vertex tree obtained by attaching $n-8$ pendent vertices to the center of the path on five vertices. Applying Lemma 3 to $G=U_{n}^{3}$ by setting *v* to be a pendent neighbor of $v_{2}$,

$$\phi\bigl(U_{n}^{3},x\bigr)=x\cdot\phi\bigl(U_{n-1}^{1},x \bigr)-x\cdot\phi\bigl(T^{(1)},x\bigr), $$

and to $G=L_{n}$ by setting *v* to be a pendent vertex in $T_{1}$ of degree three,

$$\phi(L_{n},x)=x\cdot\phi\bigl(U_{n-1}^{1},x \bigr)-x\cdot\phi\bigl(S_{n-3}(n-6,3,3),x\bigr). $$

Note that $T^{(1)}$ is a proper spanning subgraph of $S_{n-3}(n-6,3,3)$, and thus by Lemma 4, $\phi(S_{n-3}(n-6,3,3),x)<\phi(T^{(1)},x)$ for $x\ge \lambda_{1}(S_{n-3}(n-6,3,3))$, which implies that $\phi(U_{n}^{3},x)<\phi(L_{n},x)$ for $x\ge\lambda_{1}(S_{n-3}(n-6,3,3))$, *i.e.*, $\lambda_{1}(U_{n}^{3})>\lambda_{1}(L_{n})$. Thus

$$\lambda_{1}(G)\le\lambda_{1}\bigl(U_{n}^{3} \bigr) $$

for $b=3\mbox{ or }4$.

Now we are left to compare $\lambda_{1}(U_{n}^{3})$ with $\lambda_{1}(S_{n}(n-5,5,3))$. Let $T^{(2)}$ be the $(n-4)$-vertex tree obtained by attaching $n-7$ pendent vertices to an end vertex of the path on three vertices. Applying Lemma 3 to $G=U_{n}^{3}$ by setting *v* to be a pendent vertex whose unique neighbor is of degree two,

$$\phi\bigl(U_{n}^{3},x\bigr)=x\cdot\phi\bigl(S_{n-1}(n-5,4,3),x \bigr)-\phi\bigl(S_{n-2}(n-6,4,3),x\bigr), $$

and to $G=S_{n}(n-5,5,3)$ by setting *v* to be a pendent neighbor of $v_{2}$,

$$\phi\bigl(S_{n}(n-5,5,3),x\bigr)=x\cdot\phi\bigl(S_{n-1}(n-5,4,3),x \bigr)-x^{2}\cdot\phi\bigl(T^{(2)},x\bigr). $$

Note that $P_{1}\cup P_{1}\cup T^{(2)}$ is a proper spanning subgraph of $S_{n-2}(n-6,4,3)$, and thus by Lemma 4, $\phi(S_{n-2}(n-6,4,3),x)< x^{2}\cdot\phi(T^{(2)},x)$ for $x\ge \lambda_{1}(S_{n-2}(n-6,4,3))$, which implies that $\phi(U_{n}^{3},x)>\phi(S_{n}(n-5,5,3),x)$ for $x\ge \lambda_{1}(S_{n-2}(n-6,4,3))$, *i.e.*, $\lambda_{1}(U_{n}^{3})<\lambda_{1}(S_{n}(n-5,5,3))$.

Then the result follows. □

### Lemma 11

*For* $n\ge8$, $\lambda_{1}(U_{n}^{2})<\lambda_{1}(U_{n}^{1})<\lambda_{1}(S_{n}(n-4,4,3))$, *for* $n=10$, $\lambda_{1}(U_{n}^{2})<\lambda_{1}(U_{n}^{1})<\lambda_{1}(S_{n}(n-5,5,3))$, *for* $n=11$, $\lambda_{1}(U_{n}^{2})<\lambda_{1}(S_{n}(n-5,5,3))<\lambda_{1}(U_{n}^{1})$, *and for* $n\ge12$, $\lambda_{1}(S_{n}(n-5,5,3))<\lambda_{1}(U_{n}^{2})<\lambda_{1}(U_{n}^{1})$.

### Proof

Let *T* be the $(n-3)$-vertex tree obtained by attaching $n-6$ pendent vertices to an end vertex of the path on three vertices.

Applying Lemma 3 to $G=U_{n}^{1}$ by setting *v* to be a pendent vertex whose unique neighbor is of degree two,

$$\phi\bigl(U_{n}^{1},x\bigr)=x\cdot \phi \bigl(S_{n-1}(n-4,3,3),x\bigr)-\phi\bigl(S_{n-2}(n-5,3,3),x \bigr), $$

and to $G=S_{n}(n-4,4,3)$ by setting *v* to be a pendent neighbor of $v_{2}$,

$$\phi\bigl(S_{n}(n-4,4,3),x\bigr)=x\cdot\phi\bigl(S_{n-1}(n-4,3,3),x \bigr)-x\cdot\phi(T,x). $$

Note that $P_{1}\cup T$ is a proper spanning graph of $S_{n-2}(n-5,3,3)$, and thus by Lemma 4,

$$x\cdot\phi(T,x)>\phi\bigl(S_{n-2}(n-5,3,3),x\bigr) $$

for $x\ge \lambda_{1}(S_{n-2}(n-5,3,3))$, which implies that

$$\phi\bigl(U_{n}^{1},x\bigr)>\phi\bigl(S_{n}(n-4,4,3),x \bigr) $$

for $x\ge \lambda_{1}(S_{n-2}(n-5,3,3))$, *i.e.*, $\lambda_{1}(U_{n}^{1})<\lambda_{1}(S_{n}(n-4,4,3))$.

By Lemma 3, we have

$$\begin{aligned}& \phi\bigl(U_{n}^{1},x\bigr)=x^{n-8} \bigl[x^{8}-nx^{6}-2x^{5}+(4n-17)x^{4}+2x^{3}-(4n-24)x^{2}+n-7 \bigr], \\& \phi\bigl(U_{n}^{2},x\bigr)=x^{n-6} \bigl[x^{6}-nx^{4}-2x^{3}+(4n-17)x^{2}+2x-3n+17 \bigr], \\& \phi\bigl(S_{n}(n-5,5,3),x\bigr)=x^{n-6} \bigl[x^{6}-nx^{4}-2x^{3}+(5n-28)x^{2}-3n+21 \bigr], \end{aligned}$$

and thus

$$\begin{aligned}& \phi\bigl(U_{n}^{1},x\bigr)-\phi\bigl(U_{n}^{2},x \bigr)=-x^{n-8}(n-7) (x+1) (x-1), \\& \phi\bigl(U_{n}^{2},x\bigr)-\phi\bigl(S_{n}(n-5,5,3),x \bigr)=x^{n-6}(n-7)f(x), \end{aligned}$$

where

$$f(x)=(11-n)x^{2}+2x-4. $$

Obviously, $\phi(U_{n}^{1},x)<\phi(U_{n}^{2},x)$ for $x>1$, *i.e.*, $\lambda_{1}(U_{n}^{1})>\lambda_{1}(U_{n}^{2})$.

Note that $\lambda_{1}(G)>2$ if *G* is a unicyclic graph different from a cycle. If $n=10$, then

$$f(x)=x^{2}+2x-4>0 $$

for $x>2$. If $n=11$, then

$$f(x)=2x-4>0 $$

for $x>2$. If $n\ge12$, then

$$\begin{aligned} f(x) =&(11-n)x^{2}+2x-4 \\ < &(11-n)2^{2}+2\cdot2-4=4(11-n)< 0. \end{aligned}$$

Thus $f(x)>0$ for $x>2$ if $n=10,11$ and $f(x)<0$ for $x>2$ if $n\ge12$, which implies that $\lambda_{1}(U_{n}^{2})<\lambda_{1}(S_{n}(n-5,5,3))$ for $n=10,11$ and $\lambda_{1}(U_{n}^{2})>\lambda_{1}(S_{n}(n-5,5,3))$ for $n\ge12$.

By direct calculation, we have $\lambda_{1}(U_{n}^{1})<\lambda_{1}(S_{n}(n-5,5,3))$ for $n=10$ and $\lambda_{1}(U_{n}^{1})>\lambda_{1}(S_{n}(n-5,5,3))$ for $n=11$.

The result follows easily. □

Now we prove our main result in this section.

### Theorem 1

*Among the graphs in* $\mathbb{U}(n)$,

(i) $U_{n,3} \cong S_{n}(n-3,3,3)$ *for* $n\ge6$ *and* $S_{n}(n-4,4,3)$ *for* $n\ge8$ *are respectively the unique graphs with the first and the second maximum spectral radii*;
(ii) $S_{n}(n-5,5,3)$ *for* $n=10$ *and* $U_{n}^{1}$ *for* $n\ge11$ *are the unique graphs with the third maximum spectral radius*;
(iii) $S_{n}(n-5,4,4)$ *for* $n=10$, $S_{n}(n-5,5,3)$ *for* $n=11$, *and* $U_{n}^{2}$ *for* $n\ge12$ *are the unique graphs with the fourth maximum spectral radius*;
(iv) $U_{n}^{1}$ *for* $n=10$, $U_{n}^{2}$ *for* $n=11$, *and* $S_{n}(n-5,5,3)$ *for* $n\ge 12$ *are the unique graphs with the fifth maximum spectral radius*,

*and the spectral radii of* $U_{n,3} \cong S_{n}(n-3,3,3)$, $S_{n}(n-4,4,3)$, $S_{n}(n-5,5,3)$, $U_{n}^{1}$ *and* $U_{n}^{2}$ *are*, *respectively*, *the largest roots of the equations on* *x* *as follows*:

$$\begin{aligned}& x^{6}-nx^{4}-2x^{3}+(3n-12)x^{2}-n+5=0, \\& x^{6}-nx^{4}-2x^{3}+(4n-19)x^{2}-2n+12=0, \\& x^{6}-nx^{4}-2x^{3}+(5n-28)x^{2}-3n+21=0, \\& x^{8}-nx^{6}-2x^{5}+(4n-17)x^{4}+2x^{3}-(4n-24)x^{2}+n-7=0, \\& x^{6}-nx^{4}-2x^{3}+(4n-17)x^{2}+2x-3n+17=0. \end{aligned}$$

### Proof

Let $G\in\mathbb{U}(n)$ with unique cycle of length $m\ge3$, where $n \ge2m$.

If $m\ge4$, then by Lemmas 8 and 2,

$$\lambda_{1}(G)\le \lambda_{1}(U_{n,4})< \lambda_{1}\bigl(S_{n}(n-5,5,3)\bigr). $$

Suppose in the following that $m=3$. Then $G\cong C_{3}(T_{1},T_{2},T_{3})$, where $|V(T_{1})|=a-1$, $|V(T_{2})|=b-1$, $|V(T_{3})|=c-1$, $a+b+c=n+3$, $a\ge b\ge c\ge3$.

If $G \cong S_{n}(a,b,c)$, then by Lemma 9, $U_{n,3}\cong S_{n}(n-3,3,3)$, $S_{n}(n-4,4,3)$ and $S_{n}(n-5,5,3)$ are, respectively, the unique graphs with the first, the second and the third maximum spectral radii.

Suppose that $G\ncong S_{n}(a,b,c)$. If $c\ge4$, then by Lemmas 2 and 9, $\lambda_{1}(G)<\lambda_{1}(S_{n}(n-5,5,3))$. Suppose that $c=3$. If $b=3$ or 4 and $G\ncong U_{n}^{1},U_{n}^{2}$, then by Lemma 10, $\lambda_{1}(G)< \lambda_{1}(S_{n}(n-5,5,3))$. If $b \ge5$, then by Lemmas 2 and 9, $\lambda_{1}(G)<\lambda_{1}(S_{n}(n-5,5,3))$. Thus

$$\lambda_{1}(G)< \lambda_{1}\bigl(S_{n}(n-5,5,3) \bigr) $$

if $G\ncong S_{n}(n-3,3,3)$, $S_{n}(n-4,4,3)$, $S_{n}(n-5,5,3)$, $U_{n}^{1}$, $U_{n}^{2}$ for $n\ge10$.

It follows from Lemma 11 that

$$\lambda_{1}\bigl(U_{n}^{2}\bigr)< \lambda_{1}\bigl(U_{n}^{1}\bigr)< \lambda_{1}\bigl(S_{n}(n-4,4,3)\bigr) $$

for $n\ge8$, and thus (i) follows.

Suppose that $G\ncong S_{n}(n-3,3,3)$, $S_{n}(n-4,4,3)$.

Suppose that $n=10$. If $m\ge4$, then by Lemma 8 and direct calculation,

$$\lambda_{1}(G)\le \lambda_{1}(U_{10,4})< \lambda_{1}\bigl(U_{10}^{1}\bigr). $$

Suppose that $m=3$. If $G\cong S_{10}(a,b,c)$, then $G \cong S_{10}(5,5,3)$ or $S_{10}(5,4,4)$. Suppose that $G\ncong S_{10}(a,b,c), U_{10}^{1}$. If $c \ge4$, then by Lemmas 1 and 2, we may construct another graph still different from $S_{10}(a,b,c)$ and $U_{10}^{1}$ with a larger spectral radius. Suppose that $c = 3$. If $b = 3$ or 4 and $G \ncong U_{10}^{2}$, then similar to the proof of Lemma 10 and direct calculation,

$$\lambda_{1}(G)\le \lambda_{1}\bigl(U_{10}^{3} \bigr)< \lambda_{1}\bigl(U_{10}^{2}\bigr)< \lambda_{1}\bigl(U_{10}^{1}\bigr). $$

If $b \ge5$, then by Lemmas 1 and 2, we may construct another graph still different from $S_{10}(a,b,c)$ and $U_{10}^{1}$ with a larger spectral radius. So if $G \ncong S_{10}(a,b,c), U_{10}^{1}$, then

$$\lambda_{1}(G)< \lambda_{1}\bigl(U_{10}^{1} \bigr). $$

Now by direct calculation,

$$\lambda_{1}\bigl(U_{10}^{1}\bigr)< \lambda_{1}\bigl(S_{10}(5,4,4)\bigr)< \lambda_{1} \bigl(S_{10}(5,5,3)\bigr), $$

and thus the result for $n=10$ follows.

Suppose that $n=11$. If $m\ge4$, then by Lemma 8 and direct calculation,

$$\lambda_{1}(G)\le \lambda_{1}(U_{11,4})< \lambda_{1}\bigl(U_{11}^{2}\bigr). $$

Suppose that $m=3$. If $G \cong S_{11}(a,b,c)$, then $G\cong S_{11}(6,5,3)$, $S_{11}(6,4,4)$, or $S_{11}(5,5,4)$. Suppose that $G \ncong S_{11}(a,b,c)$, $U_{11}^{1}$, $U_{11}^{2}$. If $c \ge4$, then by Lemmas 1 and 2, either $\lambda_{1}(G)<\lambda_{1}(U_{11}^{2})$, or we may construct another graph still different from $S_{11}(a,b,c)$, $U_{11}^{1}$ and $U_{11}^{2}$ with a larger spectral radius. Suppose that $c = 3$. If $b = 3$ or 4, then similar to the proof of Lemma 10 and direct calculation,

$$\lambda_{1}(G)\le \lambda_{1}\bigl(U_{11}^{3} \bigr)< \lambda_{1}\bigl(U_{11}^{2}\bigr)< \lambda_{1}\bigl(U_{11}^{1}\bigr). $$

If $b \ge5$, then by Lemmas 1 and 2, either $\lambda_{1}(G)< \lambda_{1}(U_{11}^{2})$, or we may construct another graph still different from $S_{11}(a,b,c)$, $U_{11}^{1}$ and $U_{11}^{2}$ with a larger spectral radius. So if $G \ncong S_{11}(a,b,c), U_{11}^{1}, U_{11}^{2}$, then

$$\lambda_{1}(G)< \lambda_{1}\bigl(U_{11}^{2} \bigr). $$

Now by direct calculation,

$$\lambda_{1}\bigl(S_{11}(5,5,4)\bigr)< \lambda_{1} \bigl(S_{11}(6,4,4)\bigr)< \lambda _{1}\bigl(U_{11}^{2} \bigr)< \lambda_{1}\bigl(S_{11}(6,5,3)\bigr)< \lambda_{1}\bigl(U_{11}^{1}\bigr), $$

and thus the result for $n=11$ follows.

If $n\ge12$, then the result follows from Lemma 11. □

## The least eigenvalue of graphs in $\mathbb{U}(n)$

In this section, we determine the minimum least eigenvalue of graphs in $\mathbb{U}(n)$.

### Lemma 12

*Let* $G\in\mathbb{U}(n)$ *with unique cycle of length* *m*, *where* $n\ge2m\ge8$. *Then* $\lambda_{n}(G)\ge\lambda_{n}(U_{n,4})$ *with equality if and only if* $G \cong U_{n,4}$.

### Proof

If $m\ge4$, then by Lemma 8, the Perron-Frobenius theorem [1] and noting that $U_{n,4}$ is a bipartite graph, we have

$$-\lambda_{n}(G)\le \lambda_{1}(G)\le\lambda_{1}(U_{n,4})=- \lambda_{n}(U_{n,4}), $$

and thus $\lambda_{n}(G)\ge\lambda_{n}(U_{n,4})$ with equality if and only if $G \cong U_{n,4}$. □

### Lemma 13

*If* $G\cong S_{n}(a,b,c)$ *with* $a+b+c=n+3$ *and* $a\ge b\ge c\ge3$, *then* $S_{n}(n-3,3,3) \cong U_{n,3}$ *for* $n\ge6$ *is the unique graph with minimum least eigenvalue*.

### Proof

Let **x** be a unit eigenvector of $S_{n}(a,b,c)$ corresponding to $\lambda_{n}(S_{n}(a,b,c))=\lambda_{n}$.

Denote by $u_{1}$, $u_{2}$, $u_{3}$ a pendent neighbor of $v_{1}$, $v_{2}$, $v_{3}$ in $S_{n}(a,b,c)$, respectively. It is easily seen that

$$x_{u_{1}}=\frac{x_{v_{1}}}{\lambda_{n}},\qquad x_{u_{2}}=\frac{x_{v_{2}}}{\lambda_{n}}, \qquad x_{u_{3}}=\frac{x_{v_{3}}}{\lambda_{n}}. $$

Suppose that $x_{u_{2}}=0$ and $x_{v_{1}}=x_{v_{2}}$. Then $x_{v_{1}}=x_{v_{2}}=0$. Since

$$\lambda_{n}x_{v_{2}}=(b-2)x_{u_{2}}+x_{v_{1}}+x_{v_{3}}, $$

we have $x_{v_{3}}=0$, and thus $x_{u_{3}}=0$, *i.e.*, ${\mathbf{x}}=0$, which is a contradiction. Then either $x_{u_{2}}\ne0$ or $x_{v_{1}}\ne x_{v_{2}}$.

Suppose that $a>b$. If $|x_{v_{1}}|< |x_{v_{2}}|$, then by Lemma 5,

$$\lambda_{n}\bigl(S_{n}(a,b,c)\bigr)>\lambda_{n} \bigl(S_{n}(a-1,b+1,c)\bigr)>\cdots>\lambda_{n} \bigl(S_{n}(b,a,c)\bigr), $$

which is a contradiction. If $|x_{v_{1}}|\ge|x_{v_{2}}|$, then by Lemma 5(i) and noting that either $x_{u_{2}}\ne0$ or $x_{v_{1}}\ne x_{v_{2}}$,

$$\lambda_{n}\bigl(S_{n}(a,b,c)\bigr)>\lambda_{n} \bigl(S_{n}(a+1,b-1,c)\bigr). $$

If $a=b$, then whether $|x_{v_{1}}|\ge|x_{v_{2}}|$ or $|x_{v_{1}}|< |x_{v_{2}}|$, by Lemma 5(i),

$$\lambda_{n}\bigl(S_{n}(a,b,c)\bigr)>\lambda_{n} \bigl(S_{n}(a+1,b-1,c)\bigr). $$

Then

$$\lambda_{n}\bigl(S_{n}(a,b,c)\bigr)>\lambda_{n} \bigl(S_{n}(a+1,b-1,c)\bigr) $$

for $b\ge4$, and thus the result follows easily. □

### Lemma 14

*Let* $G\cong C_{3}(T_{1},T_{2},T_{3})$ *with* $a+b+c=n+3$, $|V(T_{1})|=a-1$, $|V(T_{2})|=b-1$, $|V(T_{3})|=c-1$, $a\ge b\ge c\ge3$, *and* $n\ge7$. *Suppose that* $G\ncong S_{n}(a,b,c)$. *Then* $\lambda_{n}(G)>\lambda_{n}(S_{n}(n-3,3,3))$.

### Proof

If $b\ge4$ or $c\ge4$, then by Lemmas 6 and 13,

$$\lambda_{n}(G)\ge\lambda_{n}\bigl(S_{n}(a,b,c) \bigr)>\lambda_{n}\bigl(S_{n}(n-3,3,3)\bigr). $$

Suppose that $b=c=3$. Let $r=\max\{d_{G}(u,v_{1}):u\in V(T_{1})\}$. Obviously, $r\ge2$ as $G\ncong S_{n}(a,b,c)$.

If $r\ge3$, then by Lemma 6, we may get a graph $G'\ncong S_{n}(a,b,c)$ with $r=2$ such that $\lambda_{n}(G)\ge\lambda_{n}(G')$.

Suppose that $r=2$.

If there are at least two non-pendent neighbors of $v_{1}$ outside the triangle in *G*, then by Lemma 6, we may get a graph $G''\ncong S_{n}(a,b,c)$ with exactly one non-pendent neighbor of $v_{1}$ outside the triangle in $G''$ such that $\lambda_{n}(G)\ge\lambda_{n}(G'')$.

Suppose that there is exactly one non-pendent neighbor, say *w*, of $v_{1}$ outside the triangle in *G*. Denote by $w_{1}$ a pendent neighbor of *w* in *G* and by *u* a pendent neighbor of $v_{1}$ in *G* if existed.

Let **x** be a unit eigenvector of *G* corresponding to $\lambda_{n}(G)=\lambda_{n}$.

Suppose that $x_{w_{1}}=0$ and $x_{w}=x_{v_{1}}$. Then $x_{w}=x_{v_{1}}=x_{u}=0$. It is easily seen that

$$\lambda_{n}x_{v_{1}}=x_{v_{2}}+x_{v_{3}}=0, $$

*i.e.*, $x_{v_{3}}=-x_{v_{2}}$. On the other hand, note that

$$\lambda_{n}x_{v_{2}}=\frac{x_{v_{2}}}{\lambda_{n}}+x_{v_{3}}= \frac {x_{v_{2}}}{\lambda_{n}}-x_{v_{2}}. $$

If $x_{v_{2}}=0$, then $x_{v_{3}}=0$, which implies that ${\mathbf{x}}=0$, which is a contradiction. Suppose that $x_{v_{2}}\ne0$. Then $\lambda_{n}=-\frac{1+\sqrt{5}}{2}$. Let *H* be the 5-vertex graph obtained by attaching a path on two vertices to a vertex of a triangle. Obviously, *H* is a subgraph of *G*, and thus by the interlacing theorem [1],

$$\lambda_{n}\le\lambda_{5}(H)=-1.675< -\frac{1+\sqrt{5}}{2}= \lambda_{n}, $$

which is a contradiction. Thus either $x_{w_{1}}\ne0$ or $x_{w}\ne x_{v_{1}}$.

It follows from Lemma 5(i) that whether $|x_{v_{1}}|\ge|x_{w}|$ or $|x_{v_{1}}|<|x_{w}|$, we may get $\lambda_{n}(G)>\lambda_{n}(S_{n}(n-3,3,3))$. □

Let *G* be a unicyclic graph with at least ten vertices. It was shown in [6] that $\lambda_{1}(G)<\sqrt{n}$. Recall that [1] $-\lambda_{n}(G)\le\lambda_{1}(G)$, which implies that $\lambda_{n}(G)>-\sqrt{n}$. On the other hand, by the interlacing theorem [1],

$$\lambda_{n}(G)\le\lambda_{3}(P_{3})=-\sqrt{2}. $$

Thus

$$-\sqrt{n}< \lambda_{n}(G)\le-\sqrt{2}. $$

### Theorem 2

*Among the graphs in* $\mathbb{U}(n)$, $U_{n,4}$ *for* $n=8,9,10$ *and* $U_{n,3}\cong S_{n}(n-3,3,3)$ *for* $n=6,7$ *and* $n\ge11$ *are the unique graphs with minimum least eigenvalue*, *and the least eigenvalues of* $U_{n,3}\cong S_{n}(n-3,3,3)$ *and* $U_{n,4}$ *are*, *respectively*, *the smallest roots of the equations on* *x* *as follows*:

$$\begin{aligned}& x^{6}-nx^{4}-2x^{3}+(3n-12)x^{2}-n+5=0, \\& x^{8}-nx^{6}+(5n-26)x^{4}-(5n-32)x^{2}+n-7=0. \end{aligned}$$

### Proof

Let $G\in\mathbb{U}(n)$, where $n\ge6$. The case $n=6$ is trivial. Suppose that $n\ge7$.

If the unique cycle of *G* is of length at least four, then $n\ge8$, and by Lemma 12, $\lambda_{n}(G)\ge\lambda_{n}(U_{n,4})$ with equality if and only if $G\cong U_{n,4}$.

If the unique cycle of *G* is of length three, then either $G\cong S_{n}(a,b,c)$, and thus by Lemma 13, $\lambda_{n}(G)\ge\lambda_{n}(S_{n}(n-3,3,3))$ with equality if and only if $G\cong S_{n}(n-3,3,3)$, or $G\ncong S_{n}(a,b,c)$, and thus by Lemma 14, $\lambda_{n}(G)> \lambda_{n}(S_{n}(n-3,3,3))$.

Thus $S_{n}(n-3,3,3)$ is the unique graph with minimum least eigenvalue for $n=7$, and

$$\lambda_{n}(G)\ge\min\bigl\{ \lambda_{n}(U_{n,4}), \lambda_{n}\bigl(S_{n}(n-3,3,3)\bigr)\bigr\} $$

for $n\ge8$.

We are left to compare $\lambda_{n}(U_{n,4})$ and $\lambda_{n}(S_{n}(n-3,3,3))$ for $n\ge8$. By direct calculation, we have $\lambda_{n}(U_{n,4})<\lambda_{n}(S_{n}(n-3,3,3))$ for $n=8,9,10$ and $\lambda_{n}(U_{n,4})>\lambda_{n}(S_{n}(n-3,3,3))$ for $n=11,12,13,14,15$. Suppose in the following that $n\ge16$.

Let $G_{n}$ be the *n*-vertex unicyclic graph obtained from $U_{n+1,4}$ by deleting the unique pendent neighbor of $v_{2}$.

By Lemma 1, $\lambda_{1}(G_{n})>\lambda_{1}(U_{n,4})$. Note that both $G_{n}$ and $U_{n,4}$ are bipartite graphs, and thus $\lambda_{n}(G_{n})<\lambda_{n}(U_{n,4})$.

By Lemma 3,

$$\begin{aligned}& \phi\bigl(S_{n}(n-3,3,3),x\bigr)=x^{n-6}f(x), \\& \phi(G_{n},x)=x^{n-6}g(x), \end{aligned}$$

where

$$\begin{aligned} \begin{aligned} &f(x)=x^{6}-nx^{4}-2x^{3}+(3n-12)x^{2}-n+5, \\ &g(x)=x^{6}-nx^{4}+(4n-19)x^{2}-2n+11. \end{aligned} \end{aligned}$$

Obviously, $\lambda_{n}(S_{n}(n-3,3,3))$ and $\lambda_{n}(G_{n})$ are, respectively, the smallest roots of $f(x)=0$ and $g(x)=0$.

It is easily seen that $f(x)=g(x)+h(x)$, where

$$h(x)=-2x^{3}-(n-7)x^{2}+n-6. $$

Note that

$$\begin{aligned}& h(0)=n-6>0, \\& h(-\sqrt{2})=-n+8+4\sqrt{2}< 0, \\& h(-\sqrt{n})=2n^{3/2}-n^{2}+8n-6< 0, \end{aligned}$$

and thus $h(x)<0$ for $-\sqrt{n}< x\le-\sqrt{2}$.

Let $r=\lambda_{n}(G_{n})$. Note that $-\sqrt{n}< r\le-\sqrt{2}$. It follows that $h(r)<0$, and thus

$$f(r)=g(r)+h(r)=h(r)< 0, $$

which implies that $\lambda_{n}(S_{n}(n-3,3,3))<\lambda_{n}(G_{n})$.

Now we have $\lambda_{n}(S_{n}(n-3,3,3))<\lambda_{n}(U_{n,4})$, and thus the result for $n\ge8$ follows. □

## The maximum spread of graphs in $\mathbb{U}(n)$

Now we end this paper by determining the graph with maximum spread among graphs in $\mathbb{U}(n)$.

### Theorem 3

*Among the graphs in* $\mathbb{U}(n)$, *where* $n\ge6$, $U_{n,4}$ *for* $n=8$ *and* $U_{n,3}$ *for* $n\ne8$ *are the unique graphs with maximum spread*.

### Proof

By Theorems 1 and 2, the results for $n\ne8,9,10$ follow.

Suppose in the following that $n=8,9,10$.

If the unique cycle of *G* is of length at least four, then by Lemmas 8 and 12, $s(G)\le s(U_{n,4})$ with equality if and only if $G\cong U_{n,4}$.

If the unique cycle of *G* is of length three, then by Theorem 1 and the proof of Theorem 2, $s(G)\le s(U_{n,3})$ with equality if and only if $G\cong U_{n,3}$.

Then the results for $n=8,9,10$ follow from direct calculations for $s(U_{n,3})$ and $s(U_{n,4})$. □

## Results and discussion

In this paper, we mainly focus on the spectral properties of graphs in $\mathbb{U}(n)$, including the spectral radius, the least eigenvalue, and the spread. The main results obtained are as follows:

(i) the first five maximum spectral radii among graphs in $\mathbb{U}(n)$;
(ii) the minimum least eigenvalue among graphs in $\mathbb{U}(n)$;
(iii) the maximum spread among graphs in $\mathbb{U}(n)$.

## Conclusions

The spectral radius, the least eigenvalue, and the spread are the most important spectral properties of graphs, which are also the corn of spectral graph theory. So the research for such graph-spectrum descriptors is of great importance and value.

As the simplest connected graphs, the trees and unicyclic graphs are always the focus of research in graph theory. The investigation of spectral properties of trees has a long history, and a large number of results have been established. In contrast, the research of spectral properties of unicyclic graphs is still inadequate.

In this paper, we focus on a type of unicyclic graphs, each of whose vertices on the unique cycle is of degree at least three, and establish some bounds for their spectral radii, least eigenvalues, and the spreads. In particular, we determine the first five maximum spectral radii, the minimum least eigenvalue, and the maximum spread, respectively.
